# Voclosporin: A comprehensive review of its role as a novel calcineurin inhibitor in the management of systemic lupus erythematosus

**DOI:** 10.1097/MD.0000000000042858

**Published:** 2025-06-20

**Authors:** Patrick Ashinze, Nelson Mafua, Suvam Banerjee, Eniola Obafemi, Akande Eniola, Egbunu Emmanuel, Akogwu Ocholi Edache, Chukwu Bethrand Ozioma, Andrew Awuah Wireko, Toufik Abdul-Rahman

**Affiliations:** a Faculty of Clinical Sciences, University of Ilorin, Ilorin, Nigeria; b 82 Division Medical Service Hospital, Enugu, Nigeria; c Division of Research, Toufik’s World Medical Association, Sumy, Ukraine; d The Lind League, Nigeria; e Faculty of Clinical Sciences, Madonna University, Nigeria; f Department of Health and Family Welfare, Burdwan Medical College and Hospital, Government of West Bengal, Kolkata, West Bengal, India; g Department of Clinical Services, Federal Medical Centre, Niger, Nigeria.

**Keywords:** calcineurin inhibitors, lupus nephritis, systemic lupus erythematosus, voclosporin

## Abstract

Systemic lupus erythematosus is an autoimmune disease with diverse clinical manifestations, including lupus nephritis. Calcineurin inhibitors (CNIs) are a treatment option, but traditional CNIs have limitations. Voclosporin, a novel oral CNI, inhibits calcineurin to modulate T-cell activation and stabilize podocytes in lupus nephritis. This review assesses voclosporin’s therapeutic potential in treating SLE (lupus nephritis), examining its mechanism of action, clinical efficacy, safety profile, and advantages over other CNIs. A broad search was conducted to identify studies published from 2009 to 2024 on voclosporin and other CNIs in lupus nephritis, using databases such as PUBMED, SCOPUS, Google Scholar and Cochrane Library. MeSH Keywords included “voclosporin,” “lupus nephritis,” “systemic lupus erythematosus,” and “calcineurin inhibitors.” Studies were included if they reported relevant clinical outcomes, evaluated voclosporin in lupus nephritis, or provided comparative data on voclosporin versus other CNIs, focusing on randomized controlled trials, systematic reviews, meta-analyses, retrospective studies and cohort studies. Voclosporin demonstrated higher renal response rates at 52 weeks than standard treatment alone (40.8% vs 22.5%). It has stable pharmacokinetics, reducing the need for individualized dose adjustments and frequent monitoring. Safety outcomes show a lower incidence of adverse effects like hypertension and hyperlipidemia compared to traditional CNIs. Voclosporin offers superior efficacy and safety compared to traditional CNIs for managing lupus nephritis, with predictable dosing and a favorable side effect profile. Continued research is needed to optimize voclosporin’s use and support personalized medicine approaches.

## 1. Introduction

Systemic lupus erythematosus (SLE) is an autoimmune disease of relapsing-remitting nature that often evolves with severe complications. Its chronic, intractable course has a significant impact on medical care utilization, activities of daily living, and quality of life.

In SLE, the loss of immunological tolerance against self-antigens manifests with multisystemic involvement. The condition has several phenotypes with varying clinical presentations from mild mucocutaneous manifestations to severe multiorgan involvement.^[1]^ Its exact pathogenesis is yet to be fully understood and the disease still poses significant morbidity and mortality risk.^[2]^

The disease appears particularly in young people, aged between 15 and 45 years, with a striking female predominance. There are almost 10 female patients for every male affected by SLE. Adjusted prevalence rates worldwide are approaching or even exceeding 50 to 100 per 100,000 adults.^[3]^ The Black population is more commonly affected by the disease, 2 to 3 times more than the White population.^[4]^ The discrepancies in rates between ethnic groups are in part due to genetic factors as well as environmental factors such as smoking and dietary habits.^[5]^

Systemic Lupus Erythematosus is a multifaceted autoimmune disease distinguished by its wide-ranging symptomatology, fluctuating disease activity, and episodic worsening of symptoms. Patients often initially report nonspecific systemic complaints, including persistent tiredness and recurrent low-grade fevers. Cutaneous and mucosal abnormalities, such as rashes or ulcers, are common, alongside joint pain and inflammation affecting the musculoskeletal system. A significant proportion of individuals develop hematologic irregularities, such as cytopenias, or neuropsychiatric conditions like cognitive dysfunction. Beyond these, SLE can impact virtually any organ system, leading to renal impairment, cardiovascular complications, pulmonary involvement, visual disturbances, or digestive tract issues, underscoring the condition’s systemic scope and diagnostic challenges.^[6]^

Complications in patients with SLE may occur either due to organ damage by the disease or due to the adverse effects of the medications. Disease process-related complications include accelerated atherosclerosis, end-stage renal disease, neurological deficits, skin damage, and alopecia. Several pregnancy-related complications are well known, including fetal loss, preeclampsia, eclampsia, and neonatal lupus.^[2]^

Long-term corticosteroid use, predisposition to osteoporosis, avascular necrosis, glaucoma, cataracts, weight gain, and poor control of diabetes mellitus highlight the Medication-induced complications. Long-term use of hydroxychloroquine may result in maculopathy and irreversible retinopathy, however rare. Cyclophosphamide use is associated with a significantly high risk of interstitial cystitis and bladder cancer.^[7]^

Systemic lupus erythematosus can be severe and is associated with increased mortality from a variety of causes, including disease activity, complications of treatment, and chronic comorbidities.^[8]^ Analysis showed that males, late disease onset, delayed diagnosis (diagnosis from disease onset > 1 year), baseline organ damage, and specific organ involvements predicted higher mortality. Of the 1494 patients followed up in the Asian registry cohort, the main causes of death were infection (34.6%), active disease (26.9%), cardiovascular and cerebrovascular events (5.13%), and malignancy (5.13%).^[9]^

Recent studies in China revealed the cumulative 1-, 3-, and 5-year survival rates were 98.3%, 96.9%, and 95.7%, respectively.^[9]^ Poor prognostic factors in SLE include African American ethnicity, renal disease, male sex, young age, older age at presentation, hypertension, low socioeconomic status, presence of antiphospholipid antibodies, and high overall disease activity.^[1]^

## 2. The use of calcineurin inhibitors in management of lupus

### 2.1. Existing management modalities of lupus

Systemic lupus erythematosus poses a complex challenge due to its diverse clinical manifestations and systemic nature. The current medical management of SLE is a multifaceted approach aimed at controlling disease activity, preventing organ damage, and improving overall quality of life.

#### 2.1.1. Immunosuppressive medications

Immunosuppressive drugs, including corticosteroids, form the initial backbone for managing SLE flares. Disease-modifying antirheumatic drugs (DMARDs) such as hydroxychloroquine are essential for their long-term disease-modifying effects and have shown efficacy in preventing flares.^[10,11]^

#### 2.1.2. Biologic therapies

Biologics, specifically belimumab, have emerged as targeted therapies for SLE. Belimumab, an inhibitor of B-cell activating factor (BAFF), has demonstrated efficacy in reducing disease activity and is approved for use in certain populations^[12,13]^

#### 2.1.3. Renal involvement

Renal involvement, common in SLE, necessitates tailored approaches. Immunosuppressive agents such as cyclophosphamide and mycophenolate mofetil are utilized to manage lupus nephritis, with the choice guided by disease severity.^[14,15]^

#### 2.1.4. Supportive therapies

Supportive therapies include nonsteroidal anti-inflammatory drugs (NSAIDs) for managing musculoskeletal symptoms and addressing comorbid conditions. Addressing vitamin D deficiency, often observed in SLE patients, is crucial.^[16,17]^

#### 2.1.5. Patient education and self-management

Empowering patients through education is integral. Providing information on medications, symptom recognition, and lifestyle modifications enhances patient engagement and adherence.^[18,19]^

#### 2.1.6. Monitoring and follow-up

Regular monitoring of disease activity, laboratory parameters, and potential medication side effects is crucial. This facilitates timely adjustments in treatment plans and improves long-term outcomes^.[19,20]^

The medical management of SLE involves a strategic integration of immunosuppressive medications, biologics, renal-specific interventions, supportive therapies, and patient education. A personalized and multidisciplinary approach is essential to address the heterogeneity of SLE manifestations and optimize outcomes for individuals living with this autoimmune disorder.

### 2.2. Calcineurine inhibitors and their efficacies in lupus management

Calcineurine inhibitors (CNIs) are a class of immunosuppressants used in the management of various autoimmune disorders and serve as essential components for immunosuppression in solid organ transplants. Their examples include cyclosporine, tacrolimus, voclosporin, and pimecrolimus.

Calcineurine inhibitors primarily act by competitively inhibiting the action of calcineurine. Calcineurin forms a phosphatase complex consisting of calcineurin-A and calcineurin-B subunits which bind to calmodulin and calcium respectively. This protein actively participates in numerous cellular processes and calcium-dependent pathways for signal transduction including T-cell activation.^[21]^ CNIs generally bind with high affinity to specific cytoplasmic receptors known as immunophilins, which include cyclophilin and FK-binding protein.^[22]^ A complex is then formed of drug-receptors, calcium, calmodulin, and calcineurin, which inhibits the phosphatase activity of calcineurin. This complex prevents the dephosphorylation and subsequent translocation of the nuclear factor of activated T cells (NF-AT), a nuclear component that initiates gene transcription for the formation of IL-2, IL-3, and IL-4, among other cytokine genes.^[23]^ By selectively inhibiting calcineurin, these drugs disrupt the transcription of IL-2 and other cytokines within T lymphocytes, thereby interfering with T-cell activation, proliferation, and differentiation.^[24]^ Although these agents primarily act on T-helper cells, they simultaneously inhibit T suppressor and T cytotoxic cells during the process.

In lupus T cells, reduced stability, synthesis, and expression, and enhanced degradation of CD3ζ are consistently evident.^[25]^ The resultant quantitative and functional deficiencies of CD3ζ in lupus T cells lead to a reciprocal increase in the expression of FcγR, enhancing pathway “rewiring” towards Syk stimulation, and consequential higher calcium influx into T cells compared to that triggered by the CD3ζ-ZAP-70 pathway.^[25]^ Hence, drugs that suppress T cell activation, typically calcineurin inhibitors, have been found efficacious in reducing SLE disease activity, particularly in lupus glomerulonephritis (LN).^[26]^

Discovered over 50 years ago, CNIs have been the backbone of immunosuppressive therapy in transplants, and the outstanding results of cyclosporine led to its use in a variety of autoimmune diseases. Multiple trials showed the efficacy of cyclosporine for different immune-mediated clinical entities, such as endogenous uveitis, Sjögren’s syndrome, myasthenia gravis, and Crohn disease.^[27]^ Even now, a meta-analysis of multiple clinical trials on various autoimmune diseases has shown that calcineurin inhibitors have significant clinical efficacy over other available options. In an analysis of 14 trials involving 7376 children and adults with atopic dermatitis, calcineurin inhibitors were significantly more effective than various potency topical corticosteroids.^[28]^ Another analysis of efficacy in IgA nephropathy using 7 relevant trials with 374 patients enrolled showed CNIs plus medium/low-dose steroids had a higher complete remission rate compared to therapy with steroid alone or placebo.^[29]^

A prominent concern of CNIs is dose-dependent renal damage that may escalate to chronic dysfunction, sometimes causing permanent impairment. These agents can also induce elevated blood pressure linked to narrowed kidney blood vessels. Neurologic disturbances such as tremors, migraines, medication-associated pain, and nerve damage are frequently observed. Additional risks encompass electrolyte abnormalities, liver injury, and severe infections – including opportunistic pathogens – due to heightened susceptibility from immune suppression. Long-term use further elevates the likelihood of developing certain cancers, such as lung malignancies and lymphatic system cancers, compounding the complexity of managing CNI therapy.^[30]^

While calcineurin inhibitors remain an important part of therapy in autoimmune diseases, their toxicity is a major limiting factor in clinical efficacy.

Multiple electrolyte derangements including hyperkalemia, hypomagnesemia, hypercalciuria, metabolic acidosis, and hyperuricemia may be challenging to manage for the clinician, especially in patients on long-term therapy. CNIs also have a narrow therapeutic window, with low levels increasing the risk of acute allograft rejection and treatment failure while high levels increase the risk of nephrotoxicity that may lead to acute or chronic kidney or kidney allograft injury.^[31]^ CNI-based therapy is also complicated by the absence of standardized dosing and the need for drug-level monitoring, and meticulous dose adjustments as well as by pharmacogenetic differences.^[32]^

### 2.3. The use of voclosporin in lupus

**Figure 1.**
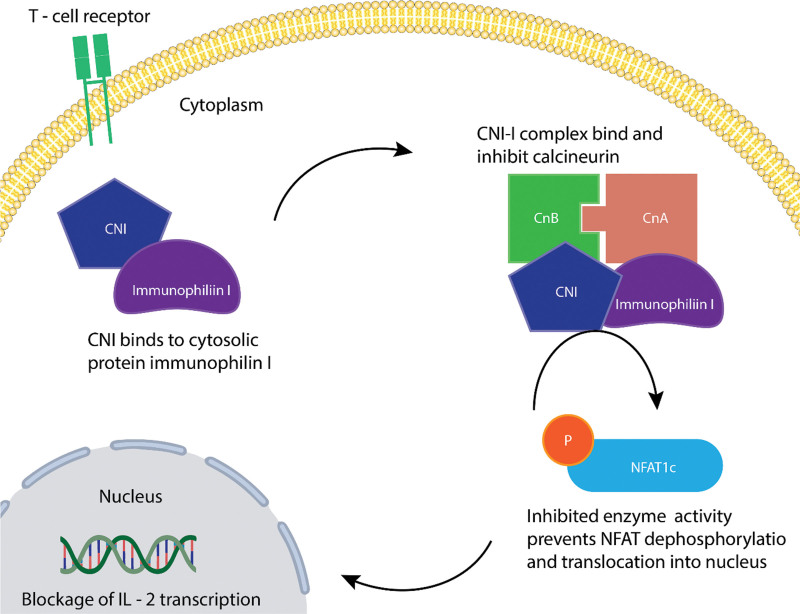
Schematic illustration of Voclosporin mechanism in the management of SLE. SLE = systemic lupus erythematosus.

Voclosporin is a novel oral calcineurin inhibitor that has gained worldwide recognition for the treatment of adult lupus nephritis. It is a semisynthetic analogue of cyclosporine that acts by inhibiting calcineurin, an enzyme that activates T cells in the immune system, via the calcineurin signal transduction pathway (Fig. 1). The overall action leads to an immunosuppressant effect that is key in its use. It has 2 distinct activities in the treatment of Lupus nephritis, immunomodulatory effects of T cells and stabilization of podocytes. In the T-cell, inhibition of calcineurin prevents the translocation of nuclear factor of activated T cells (NF-AT) to the nucleus with the subsequent reduction in the transcription of genes encoding inflammatory cytokines, resulting in the reduction of lymphocyte proliferation and T cell mediated responses. In the podocyte, the inhibition of calcineurin prevents the dephosphorylation of synaptopodin, therefore maintaining the stabilizing function of the cytoskeleton and reducing proteinuria.^[33]^ Voclosporin is 97% bound to protein and has a peak plasma time of 1.5 hours on an empty stomach and reaches its steady state at 6 days after twice daily dosing. It is predominantly metabolized by cytochrome CYP3A4 enzyme and excreted majorly in the faces. Its half-life is 30 hours^.[34,35]^ Due to the unique structure of voclosporin as compared to its parent molecule cyclosporine, it no longer needs constant drug-level monitoring as the risk for potentially dangerous side effects of nephrotoxicity has been reduced.^[36]^ Voclosporin received its first approval from the Food and Drug Administration in the USA on January 21, 2021, for the treatment of active biopsy-proven lupus nephritis (classes III–V) in adults, in conjunction with a background immunosuppressive regimen.^[37,38]^

### 2.4. Efficacy outcomes and statistical analysis

In a post hoc analysis of the phase 3 AURORA 1 clinical trial, a double-blinded placebo-controlled study, which evaluated the efficacy and safety of Voclopsorin in a subgroup of 148 participants with proliferative lupus nephritis, it was shown that participants treated with voclosporin achieved significantly higher rates of complete renal response at 12 months (34%) than participants treated with placebo (11%) when administered in combination with mycophenolate mofetil (MMF) and low-dose glucocorticoids. In addition, participants treated with voclosporin also demonstrated significantly higher rates of partial renal response at 6 months (74% vs 47%).^[39]^ AURORA 2 trial aimed to follow-up treated patients under AURORA 1 for a period of 3 years. An interim analysis of 116 patients in the voclosporin arm and 100 patients in the control arm, of whom 90 and 78, respectively, had received 30 months of total treatment (12 months in AURORA 1 and a further 18 months in AURORA 2), showed that the meaningful reductions in proteinuria seen in AURORA 1 were maintained over 30 months of treatment.^[33]^ Studies have effectively highlighted the efficacy of voclosporin over other calcineurin inhibitors, however, it will be important to evaluate the long-term efficacy in lupus nephritis.

## 3. Voclosporin clinical trials and studies

### 3.1. Assessment of safety profile

Voclosporin, a novel calcineurin inhibitor, underwent rigorous safety profile assessment in clinical trials (Table 1). In a Phase 3 trial for lupus nephritis, voclosporin demonstrated a favorable safety profile comparable to standard care, with adverse events consistent with mycophenolate mofetil (MMF) and low-dose steroids.^[40]^ This pivotal study emphasized the drug’s tolerability in treating lupus nephritis. Safety evaluation extended to a Phase 2b trial in psoriasis patients, where voclosporin exhibited a favorable safety profile, affirming its potential in dermatological conditions.^[41]^ Routine monitoring encompassed adverse events and laboratory parameters, ensuring a comprehensive understanding of voclosporin’s safety. These trials collectively position voclosporin as a well-tolerated calcineurin inhibitor. The favorable safety outcomes observed in lupus nephritis and psoriasis trials underscore its potential as a safe and effective therapeutic option, offering optimism for improved treatment modalities in conditions requiring immunosuppression.

**Table 1:** The above table provides an overview of all the known clinical trials involving Voclosporin.

Trial name	Trial phase	Trial status	Purpose/objective	Participants	Indication	Results/outcome
N/A	Phase 1	Completed	Demonstrated safety profile, established dosing regimen1	Healthy volunteers	Safety, pharmacokinetics	N/A
AURA-LV (AURA)2	Phase 2	Completed	Assess efficacy	265 patients	Lupus nephritis	Positive results, met primary endpoint of complete remission in a significantly higher percentage of patients compared to control group
AURORA (AURORA 1)3	Phase 3	Completed	Evaluate safety and efficacy	357 patients	Lupus nephritis	Met primary endpoint of complete renal response, showed significant improvements in renal response and overall disease activity
AURORA (AURORA 2)	Phase 3	Completed	Evaluated the long-term safety, tolerability, and efficacy of voclosporin compared to placebo in patients with lupus nephritis receiving an additional 2 years of treatment following completion of the 1-year AURORA 1 study	216 patients	Lupus nephritis	Provides further support for the use of voclosporin with mycophenolate mofetil and low-dose glucocorticoids for the treatment of lupus nephritis

AURORA = a clinical trial designation; specific phases identified (AURORA 1, AURORA 2, etc).

### 3.2. Comparison with other calcineurin inhibitors or standard treatments

In comparison to other calcineurin inhibitors (CNIs) such as cyclosporine and tacrolimus, voclosporin demonstrates enhanced efficacy and a more favorable safety profile for lupus nephritis. Voclosporin’s molecular structure provides greater stability and potency at lower doses, minimizing the risk of nephrotoxicity – a notable adverse effect seen with traditional CNIs.^[40]^

Clinical trials have illustrated voclosporin’s promising outcomes in lupus nephritis patients. In the AURORA Phase 3 trial, voclosporin combined with standard-of-care therapy (mycophenolate mofetil and corticosteroids) led to significantly higher renal response rates at 52 weeks (40.8%) compared to standard treatment alone (22.5%), with sustained benefits noted in kidney function at 104 weeks.^[37]^ This superior efficacy is linked to voclosporin’s consistent bioavailability and predictable pharmacokinetics, enhancing therapeutic outcomes without the need for individualized dose adjustments, which are commonly required with other CNIs like tacrolimus and cyclosporine^.[37,42]^

Regarding safety, voclosporin is generally better tolerated than traditional CNIs, showing a reduced incidence of side effects such as hypertension and hyperlipidemia, which are frequently associated with cyclosporine and tacrolimus.^[37,38]^

Patients on voclosporin report fewer adverse reactions related to cardiovascular and metabolic health, improving overall quality of life and potentially reducing comorbidities often seen in long-term immunosuppressive therapy. While both cyclosporine and tacrolimus are associated with increased risks of diabetes and neurotoxicity, voclosporin’s modified molecular structure contributes to a decreased risk of these complications.

Patient adherence is another area where voclosporin may hold an advantage. The simplified dosing regimen and reduced need for frequent monitoring may improve adherence compared to cyclosporine and tacrolimus, where blood levels must be closely monitored due to their narrow therapeutic windows.^[40]^ Voclosporin’s stable pharmacokinetics allow for predictable dosing, which can be a crucial factor in adherence for patients with lupus nephritis, who often require complex medication regimens. Overall, voclosporin’s balance of efficacy, tolerability, and convenience suggests it could be a significant advancement over traditional CNIs for managing autoimmune kidney conditions such as lupus nephritis.^[42]^

This suggests that voclosporin thus offers advantages over traditional calcineurin inhibitors in certain patient populations.

### 3.3. Implications and existing successors

The implications of voclosporin’s clinical trials extend beyond its immediate applications. The drug’s success in lupus nephritis and psoriasis trials opens avenues for exploration in other autoimmune diseases, broadening its potential impact across diverse medical fields.^[42]^ Additionally, the favorable safety profile positions voclosporin as a promising candidate for combination therapies, further enhancing its versatility in managing complex medical conditions.^[43]^ The comparison with standard treatments is crucial in establishing voclosporin’s position in the treatment algorithm. In lupus nephritis, where standard care involves MMF and steroids, voclosporin’s efficacy and safety offer a compelling argument for its consideration as a primary or adjunctive therapy. Understanding its comparative advantages aids clinicians in making informed decisions tailored to individual patient needs. In the context of existing successors, voclosporin’s emergence prompts considerations about its role in therapeutic landscapes. For instance, tacrolimus has long been a cornerstone in immunosuppression; however, the advent of voclosporin introduces a potential alternative with improved outcomes in specific indications. This raises questions about the future balance of utilization between established treatments and innovative options like voclosporin.

In conclusion, voclosporin’s clinical trials have provided valuable insights into its safety profile, efficacy compared to other calcineurin inhibitors, and potential implications in diverse therapeutic landscapes. The data generated from these studies contribute to the ongoing evolution of immunosuppressive therapies, with voclosporin emerging as a promising successor.^[33]^ As clinicians navigate treatment decisions, the comprehensive evaluation of voclosporin’s performance in clinical trials serves as a cornerstone for informed and personalized patient care.

### 3.4. Ongoing studies

AURORA 3: This is a phase 4 trial and a 5-year cohort of about 200 patients who have completed AURORA 1 or AURORA 2. It aims to study the long-term safety and efficacy of these patients.^[44,45]^ Its use, however, is in combination with a background immunosuppressive therapy regimen for adults with active lupus nephritis.^[45,46]^

AURORA 2: An extension study of the phase 3 AURORA 1 trial whose interim analysis has shown that voclosporin maintained significant reductions in proteinuria and stable kidney functions for 2 years.^[47]^

AURORA COVID-19: A cohort of about 200 patients in a phase 2 trial that examines the potential of voclosporin to prevent acute kidney injury and other complications in kidney transplant patients who have contracted covid-19.^[48]^

## 4. Future perspectives

Aurinia Pharmaceuticals developed oral Voclosporin which was first approved in January 2021 by the FDA for treating active Lupus Nephritis in adults following positive results from pivotal Phase II and III clinical trials.^[44]^ Some future perspectives on its use include: long-term efficacy and safety versus low-dose cyclosporin or tacrolimus, expanded treatment options and providing alternatives to existing therapies, understanding the synergistic effects and optimal dosing regimens in combination therapies, the optimal timing to start voclosporin therapy (an ongoing research), and patient stratification to identify specific patient subgroups that benefit most from voclosporin will be essential, e.g., the effects of low-dose voclosporin in patients with reduced kidney function and an approved eGFR cutoff for voclosporin contraindications are yet unknown^[47]^

### 4.1. Potential improvements or modifications in SLE treatments

Voclosporin at best increases the armamentarium of choices available for treating SLE and is not a silver bullet. Thus, here are a few suggestions for potential improvement and or modifications in SLE treatment:

a) Developing precision and personalized medicine approaches that take into account the genetic, epigenetic, molecular, and immunological differences between patients and subtypes of SLE.^[49,50]^
b) Mapping out new targets and pathways for drug development, such as B cells, T cells, cytokines, complement, toll-like receptors, and type 1 interferons.^[48,50]^
c) Examining the safety and efficacy of new or repurposed drugs such as anifrolumab, ustekinumab, baricitinib, and hydroxychloroquine for SLE.^[49,50]^
d) Making guided treatment decisions and monitoring disease outcomes using well-defined clear and consistent criteria for disease activity, remission, flare, and damage in SLE.^[49,50]^
e) and of course, improving the design and conduct of clinical trials in SLE using appropriate endpoints, biomarkers, patient-reported outcomes, and subgroup analysis.^[49,50]^
f) enhancing coordinated multidisciplinary and holistic management of SLE and the comorbidities.^[50]^

## 5. Conclusion

Voclosporin is a novel oral immunosuppressant drug in the management of SLE, a chronic relapsing autoimmune disease. A calcineurin Inhibitor, it is also a backbone of immunosuppressive therapy in organ transplants and a plethora of other autoimmune diseases.^[27]^ Being a semisynthetic analogue of its parent drug, cyclosporine, it modulates its immunosuppressant effect via 2 distinct pathways; Immunomodulatory action on T cells and Podocyte stabilization. The high potency of voclosporin, together with its favorable metabolic profile, allows the achievement of efficacy in SLE at a dose that is associated with a relatively low level of calcineurin inhibition. Potentially, this explains the lower incidence of off-target effects and a relatively improved safety profile.^[50]^

## Acknowledgments

The authors would like to acknowledge THE LIND LEAGUE and Toufik World Medical Association, Nigeria for providing the invaluable resources to kick start, culminate and leverage this research project while also enabling our capacities.

## Author contributions

**Conceptualization:** Patrick Ashinze.

**Supervision:** Toufik Abdul-Rahman.

**Writing – original draft:** Patrick Ashinze, Nelson Mafua, Suvam Banerjee, Eniola Obafemi, Akande Eniola, Egbunu Emmanuel, Akogwu Ocholi Edache, Chukwu Bethrand Ozioma.

**Writing – review & editing:** Andrew Awuah Wireko, Toufik Abdul-Rahman.
